# Leveraging Naturalistic Driving Digital Biomarkers for Early Mild Cognitive Impairment Detection: Deep Learning Strategies

**DOI:** 10.2196/83622

**Published:** 2026-03-06

**Authors:** Firas Al-Hindawi, Teresa Wu, Yutong Wen, Peter Serhan, Erica Forzani, Francis Tsow, Yonas E Geda

**Affiliations:** 1 Industrial & Systems Engineering Department King Fahd University of Petroleum and Minerals Dhahran Saudi Arabia; 2 Interdisciplinary Research Center for Smart Mobility and Logistics King Fahd University of Petroleum and Minerals Dhahran Saudi Arabia; 3 School of Computing and Augmented Intelligence Arizona State University Tempe, AZ United States; 4 ASU Mayo Center for Innovative Imaging Arizona State University Tempe, AZ United States; 5 School of Electrical, Computer and Energy Engineering Arizona State University Tempe, AZ United States; 6 Center for Bioelectronics and Biosensors Biodesign Institute Arizona State University Tempe, AZ United States; 7 School of Engineering for Matter, Transport and Energy Arizona State University Tempe, AZ United States; 8 TF Health Corporation Phoenix, AZ United States; 9 Barrow Neurological Institute Phoenix, AZ United States

**Keywords:** aging, Alzheimer’s disease, mild cognitive impairment, smart driving, machine learning, deep learning, data fusion

## Abstract

**Background:**

Alzheimer disease and related dementias are increasing worldwide, with early detection during the mild cognitive impairment (MCI) stage critical for timely intervention. Driving behavior, which reflects everyday cognitive functioning, has emerged as a promising, noninvasive, and inexpensive digital biomarker when paired with machine learning. However, prior research has often relied on controlled settings, high-level features, or assumptions that fail to capture the sporadic nature of MCI, leaving a gap in modeling naturalistic driving data for robust early detection.

**Objective:**

This study aims to address the limitations of prior work by developing deep learning strategies that leverage driving data collected in a naturalistic setting as digital biomarkers for early detection of MCI.

**Methods:**

Clinically classified participants (8 with MCI and 14 cognitively normal; N=22) drove their personal vehicles under naturalistic conditions for several consecutive days. A total of 3 participants (2 cognitively normal and 1 MCI) withdrew before completing the experiments. In-vehicle sensors recorded GPS, accelerometer, and gyroscope signals, which were segmented into full trips and turning maneuvers. Three modeling strategies were compared: (1) single-view, (2) feature-level fusion, and (3) model-level late fusion. Classification models were trained and evaluated to assess their accuracy, discriminative ability, and participant-level performance.

**Results:**

Models using full-trip data consistently outperformed turn-only inputs, with the best-performing model achieving 78% accuracy and an area under the receiver operating characteristic curve of 77%. Turn-based inputs alone demonstrated limited discriminative power; however, combining them with trip data through late fusion improved performance, though not beyond the full-trip baseline. Participant-level analysis indicated that classification accuracy improved with increased data volume, and trip-wise modeling more effectively captured the episodic nature of MCI than majority-vote aggregation. A frequency-based risk score was proposed as an interpretable and flexible output, enabling practical application in clinical and community settings.

**Conclusions:**

Naturalistic driving behavior offers a scalable and noninvasive approach for early cognitive screening. Deep learning models using full-trip naturalistic driving data show promise for detecting MCI, with fusion strategies providing supplementary insights. This framework supports proactive, real-world monitoring of cognitive decline, laying the foundation for digital health interventions in dementia prevention.

## Introduction

Alzheimer disease (AD) and AD-related dementias are a growing public health concern. In the United States, cases are projected to reach 13.2 million by 2050, placing a significant strain on health care systems and caregivers [1-6]. Worldwide, over 55 million people are affected, with numbers expected to rise to 152 million by 2050 [5,7-9]. The disease progresses gradually, beginning with an asymptomatic phase, followed by mild cognitive impairment (MCI) and eventually dementia [10]. MCI is a transitional stage between normal aging and dementia, involving cognitive but not functional decline [11,12]. Accurate diagnosis at this stage is vital, as early detection allows timely intervention to slow progression and manage symptoms [13-16].

Identifying MCI early is challenging. Current tools, such as neuropsychological tests or biomarkers such as magnetic resonance imaging and positron emission tomography, are clinic-bound, offer only brief cognitive snapshots, and could be invasive, costly, or impractical for large-scale screening [5,6,17]. These methods often miss early MCI, as markers appear later in disease progression [18], highlighting the need for noninvasive, cost-effective biomarkers that support frequent monitoring in real-life settings [5,6,17,19-22]. Recently, spatial navigation deficits and driving behavior have emerged as potential early biomarkers for preclinical AD [23,24]. Tasks such as driving engage multiple cognitive functions in real-world contexts, and even mild impairments can lead to detectable changes in driving patterns [5,6,19,25]. Research shows that older adults with preclinical AD or MCI may limit their driving range, make more navigation errors, and drive more cautiously than cognitively normal peers [25,26]. These driving changes have been linked to AD biomarkers such as elevated amyloid and tau, even when standard cognitive tests are normal [25,27,28]. Notably, such changes can be detected by machine learning (ML) models using in-vehicle sensor data, as demonstrated in studies such as LongROAD [25] and GPS driving [19]. These studies show that ML can accurately identify individuals with MCI or preclinical AD by analyzing patterns in driving behavior such as speed, acceleration, and route choice, often outperforming conventional tools [5,6,19,25], while showing potential for real-time cognitive monitoring through everyday driving.

Despite encouraging progress, ML-based driving diagnostic systems still face key challenges. Many studies rely on simple, high-level features such as trip frequency and duration [19,25], overlooking nuanced indicators such as turn velocity, acceleration patterns, and path deviations that more directly reflect cognitive function. Furthermore, monthly driving records are treated as independent, even when from the same driver, risking data leakage and inflating performance by allowing driver-specific patterns to appear in both training and testing sets. Although recent work has incorporated richer features such as metabolic rate and ensured proper train-test separation [5,6], those studies were conducted in controlled settings with predefined routes, limiting real-world applicability. Additionally, most approaches rely on summary statistics and classical ML models, missing the potential of more powerful deep learning time-series classification techniques. Finally, conventional evaluation frameworks assume MCI symptoms appear consistently across trips, whereas studies have shown that cognitive performance in MCI can vary markedly from day to day, highlighting its sporadic nature [29-31]. Individuals with MCI may only show impairment in a minority of trips, leading models to misclassify them as cognitively normal, yet even occasional MCI-like trips could still serve as an early indicator of cognitive decline.

To address these limitations, this study proposes a new framework that improves both data representation and model evaluation. It shifts from artificial test settings to naturalistic driving by equipping participants’ personal vehicles with tracking devices and allowing several days of normal driving, capturing real-world cognitive function. Instead of treating each participant’s data as a uniform whole and relying on majority-based classification, the model adopts a granular, context-aware approach, evaluating each trip and turn individually to detect episodic impairments, consistent with the sporadic nature of MCI. Rather than delivering a binary diagnosis, it identifies subtle changes over time and flags individuals for further evaluation, enabling proactive cognitive health monitoring. The framework aggregates binary trip- and turn-level classifications over time to enable longitudinal monitoring and transforms them into a continuous, participant-level risk score (eg, the proportion of trips flagged as impaired). This frequency-based risk score provides a nonbinary, dynamic measure that reflects gradual cognitive change and supports proactive follow-up rather than a 1-time diagnostic label. Specifically, the framework includes a targeted feature-extraction pipeline that segments time-series data from full trips and cognitively demanding turning maneuvers, producing 2 complementary multivariate inputs. Deep learning architectures are then evaluated under 3 paradigms: (experiment 1) single-view modeling of trips and turns, (experiment 2) feature-level fusion, and (experiment 3) model-level late fusion using dual encoders. This design is guided by 3 key research questions.

For research question 1 (RQ1), effectiveness in naturalistic settings, can driving-based digital-biomarker strategies that showed promise in previous controlled environments [5,6] remain effective amid the noise and variability of day-to-day, real-world driving?

For research question 2 (RQ2), data handling and representation, what preprocessing and feature-extraction pipeline best transforms raw multisensor driving streams into informative inputs for deep learning?

For research question 3 (RQ3), optimal modeling strategy under data constraints, given current dataset limitations, but also anticipating richer data in future studies, which learning paradigm offers the most robust path forward? To answer this, we decompose this question into three comparative subquestions:

1. Single-view baselines: How well can early MCI be detected when trips and turns are modeled independently?
2. Early (data-level) fusion: Does combining trip and turn data at the feature level improve over the best single-view model?
3. Late (model-level) fusion: Can merging latent representations from trip and turn encoders outperform both single-view and early-fusion models?

The remainder of this paper is organized as follows. The Methods section describes this study’s design, materials, and methods used to develop the framework, and the experimental methodology. The Results section presents the outcomes of the conducted experiments. The Discussion section discusses the findings and concludes the paper.

## Methods

### Study Design

**Figure 1:**
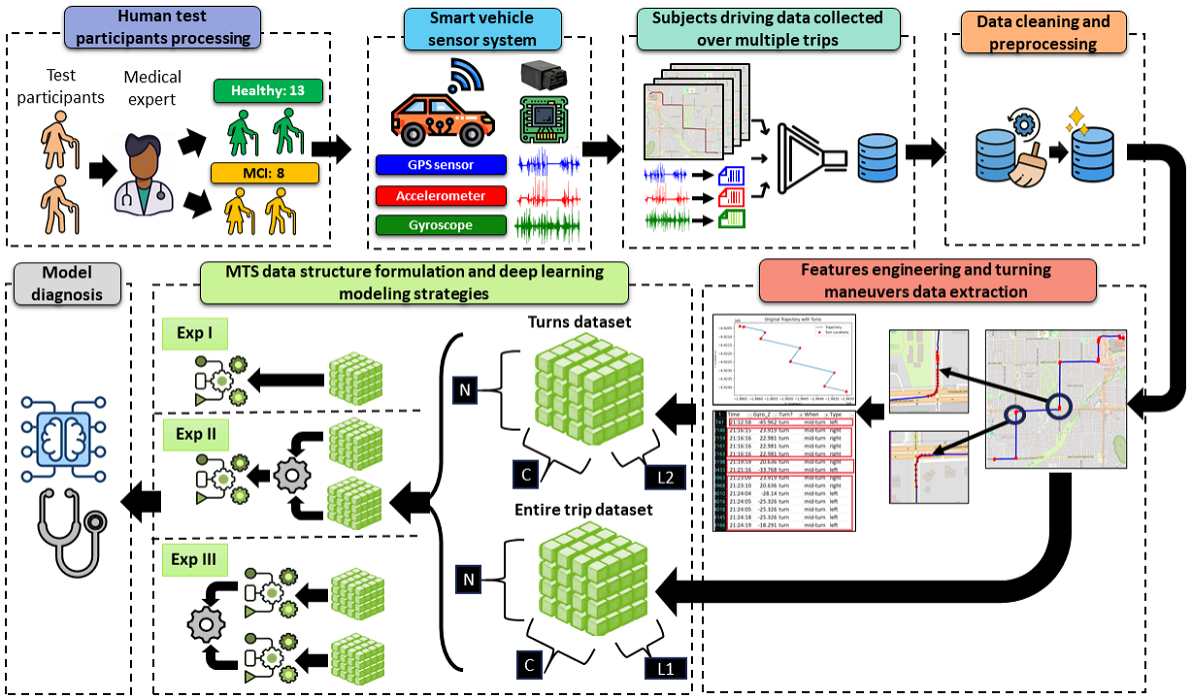
Overview of the adopted methodology, illustrating the sequential steps from participant recruitment to model training and evaluation. N denotes the number of samples (trips or turns), C the number of sensor channels, and L₁ and L₂ the interpolated sequence lengths for the trip and turn time-series inputs, respectively. MCI: mild cognitive impairment; MTS: multivariate time series.

Figure 1 presents an overview of the proposed methodology, which includes participant recruitment with expert cognitive classification, sensor-based driving data collection, preprocessing, feature engineering, and model training and evaluation. Raw sensor streams were cleaned, synchronized, and segmented into 2 complementary multivariate time-series datasets: full-trip sequences and turning maneuvers, used as inputs for subsequent experiments.

### Study Participants

To ensure accurate data interpretation, participant diagnoses were conducted in collaboration with a neurologist specializing in AD. Based on cognitive assessments, 13 participants were classified as cognitively healthy and 8 as having MCI. This grouping enabled structured comparison of driving behavior between cognitively normal and impaired individuals. Table 1 summarizes the demographic and medical diagnoses of the participants.

**Table 1:** Participant demographics and group classification: this study included 21 older adults (ages 65-85 y), with 11 male and 10 female participants. Medical evaluations classified 13 as cognitively healthy and 8 as having MCI^a^.

Participant numbers	Age (y)	Sex	Clinical condition
Participant 1	78	Male	MCI
Participant 2	80	Male	Healthy
Participant 3	70	Male	Healthy
Participant 4^b^	72	Female	Healthy
Participant 5	73	Female	Healthy
Participant 6	74	Male	MCI
Participant 7	77	Female	Healthy
Participant 8	87	Female	Healthy
Participant 9	85	Female	Healthy
Participant 10	82	Female	MCI
Participant 11	71	Female	Healthy
Participant 12	72	Female	MCI
Participant 13	65	Male	MCI
Participant 14^b^	75	Male	Healthy
Participant 15^b^	71	Female	MCI
Participant 16	76	Male	Healthy
Participant 17	75	Female	MCI
Participant 18	83	Male	Healthy
Participant 19	76	Male	MCI
Participant 20	80	Female	Healthy
Participant 21	79	Male	Healthy
Participant 22	75	Male	Healthy

^a^MCI: mild cognitive impairment.

^b^Participants withdrew before completing the experiment, leaving insufficient data for their inclusion in the final analysis.

As shown in the table, this study focused on naturalistic driving performance in adults aged 65-85 years, a group at higher risk for cognitive decline. Further, 21 participants were initially recruited, but 3 participants (participants 4, 14, and 15) withdrew before completing the driving experiment, leaving insufficient data for analysis.

### Smart Vehicle Telematics System

**Figure 2:**
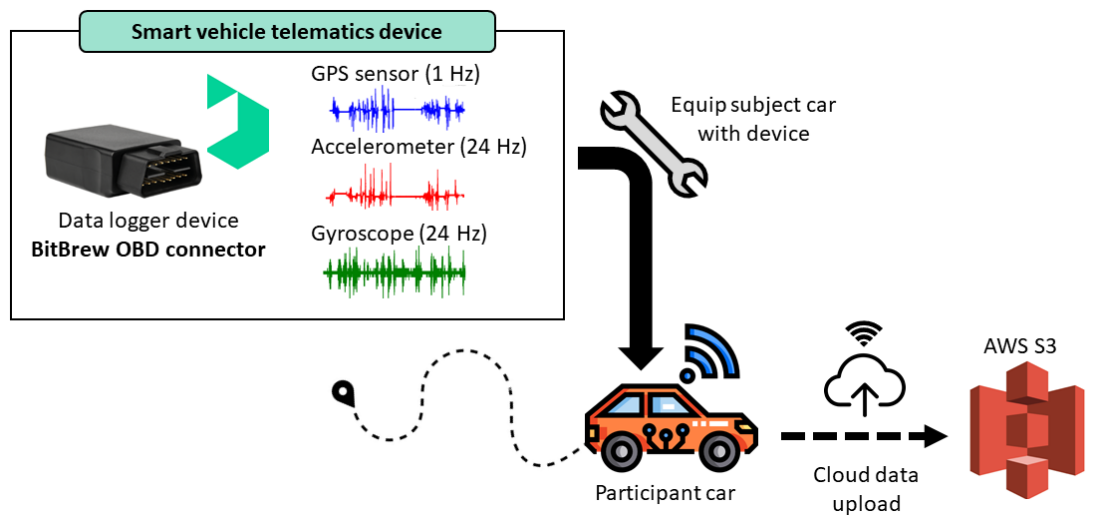
Overview of the data collection system used in the naturalistic driving study. AWS S3: Amazon Web Services Simple Storage Service; OBD: on-board diagnostics.

Each participant’s personal vehicle was fitted with a BitBrew on-board diagnostics (OBD) data logger to continuously record naturalistic driving behavior (Figure 2). This advanced telematics device integrates 3 sensors: GPS, accelerometer, and gyroscope. Together, they capture vehicle location, movement, and dynamic behavior, enabling comprehensive real-world driving analysis.

The GPS module tracks the vehicle’s location and speed, recording longitude and latitude coordinates and measuring speed in meters per second (m/s) at a frequency of 1 Hz (1 update per second). The accelerometer captures linear acceleration along 3 axes: longitudinal (forward and backward), lateral (side to side), and vertical (up and down), in m/s². It detects events such as harsh braking, rapid acceleration, and sharp turns, operating at 24 Hz for high-resolution motion detection. The gyroscope measures angular velocity in radians per second (rad/s) around 3 axes: pitch (tilting forward and backward), roll (tilting side to side), and yaw (rotational movement around the vertical axis). This sensor operates at 24 Hz and provides data on lane changes, skidding, and rollovers.

Participants drove their vehicles freely with no specific instructions, allowing for natural capture of daily driving behavior. Table 2 summarizes the driving periods for each participant, including start and end dates and total duration. All data from the OBD logger was transmitted to an AWS S3 (Amazon Web Services Simple Storage Service) bucket for cloud-based storage and analysis.

**Table 2:** Naturalistic driving test periods for study participants, detailing the start and end dates along with the duration (in days) for each observation period.

Participant number	Start date	End date	Duration (d)
Participant 1	March 29, 2023	April 7, 2023	9
Participant 2	April 13, 2023	April 20, 2023	7
Participant 3	May 4, 2023	May 11, 2023	7
Participant 4	—^a^	—	—
Participant 5	May 19, 2023	May 26, 2023	7
Participant 6	May 26, 2023	June 2, 2023	7
Participant 7	June 2, 2023	June 9, 2023	7
Participant 8	June 30, 2023	July 7, 2023	7
Participant 9	July 7, 2023	July 14, 2023	7
Participant 10	July 14, 2023	July 21, 2023	7
Participant 11	July 21, 2023	July 28, 2023	7
Participant 12	August 15, 2023	August 21, 2023	6
Participant 13	September 15, 2023	September 22, 2023	7
Participant 14	—	—	—
Participant 15	—	—	—
Participant 16	August 25, 2023	September 8, 2023	14
Participant 17	August 25, 2023	September 8, 2023	14
Participant 18	September 22, 2023	September 29, 2023	7
Participant 19	September 29, 2023	October 6, 2023	7
Participant 20^b^	October 10, 2023	January 20, 2024	86
Participant 21	October 10, 2023	October 27, 2023	17
Participant 22	October 27, 2023	November 3, 2023	7

^a^Not available.

^b^Participants did the experiment over 2 periods (October 10, 2023, to October 27, 2023, and November 12, 2023, to January 20, 2024).

### Naturalistic Driving Data Collection, Cleaning, and Preprocessing

This naturalistic driving study was conducted in Tempe, Arizona, and extended to nearby cities, including Phoenix, Glendale, Chandler, Scottsdale, and Mesa. Participants drove their personal vehicles, each equipped with a BitBrew OBD data logger, which passively recorded real-world driving behavior. The system continuously captured GPS location, accelerometer, and gyroscope data during each session, enabling a comprehensive analysis of driving patterns without imposing specific instructions or restrictions.

Recorded routes spanned a wide range of road types such as residential streets, highways, and urban roads. The dataset included a variable number of trips per participant, with differences in trip length, duration, and driving conditions reflecting the complexity of real-world behavior. However, sensor data was not always consistent across modalities for the same participant due to device malfunctions, intermittent failures, or temporary disconnections. These issues sometimes caused sensors to stop mid-trip, fail to initiate, or record out of sync, resulting in incomplete or misaligned data. To address these challenges, a structured preprocessing pipeline was applied to ensure that only high-quality, reliable, and synchronized driving records were retained for subsequent analysis.

The first step in preprocessing involved segmenting individual trips from the continuous sensor data stream. Timestamps from the accelerometer, GPS, and gyroscope were analyzed to identify gaps between recordings. If the time difference between consecutive points was less than 1 minute, the data was treated as part of the same trip. Gaps exceeding 1 minute marked the start of a new trip. After segmentation, the data were organized into participant-specific folders, with separate subfolders for each sensor and individual CSV files representing distinct trips.

Although segmentation provided structure, several data quality issues emerged. Sensor malfunctions led to missing data, and differences in recording frequency created synchronization challenges. The accelerometer and gyroscope recorded at 24 Hz, while the GPS operated at 1 Hz, resulting in mismatched data lengths. To resolve this, a cleaning process was applied. A mismatch check flagged any case where the number of trips differed across sensors. When discrepancies were found, the system logged debugging information and excluded incomplete trips. Data from all sensors was merged using shared time attributes such as day, month, hour, and minute. Trips with missing values were flagged, stored separately, and excluded from further analysis. Only complete and synchronized trips were retained. Table 3 shows the number of trips kept for each participant after preprocessing.

**Table 3:** Number of trips retained per participant after data cleaning and synchronization. Due to sensor malfunctions, missing data, and synchronization issues, the total number of trips was reduced.

Participant number	Number of trips recorded by each sensor		
	Accelerometer	GPS	Gyroscope
Participant 1	28	28	28
Participant 2	18	18	18
Participant 3	30	30	30
Participant 4	—^a^	—	—
Participant 5	46	46	46
Participant 6	21	21	21
Participant 7	39	39	39
Participant 8	6	6	6
Participant 9	11	11	11
Participant 10	11	11	11
Participant 11	21	21	21
Participant 12	3	3	3
Participant 13	13	13	13
Participant 14	—	—	—
Participant 15	—	—	—
Participant 16	10	10	10
Participant 17	11	11	11
Participant 18	11	11	11
Participant 19	4	4	4
Participant 20	126	126	126
Participant 21	6	4	6
Participant 22	24	24	24

^a^Not available.

### Features, Engineering, and Turning Maneuvers Data Extraction

#### Turning Maneuvers Data Extraction

Turning a vehicle is a cognitively demanding task involving decision-making, divided attention, working memory, processing speed, and spatial orientation. These functions are often impaired in individuals with MCI and may serve as early biomarkers for AD. Following prior studies [5,6], this work focuses on turning maneuvers, which have shown potential in detecting cognitive decline in standardized controlled experiments. Unlike previous research, this study uses naturalistic driving, where participants follow their routine behavior without instructions. This introduces additional noise into the data, allowing us to test whether turning-related features remain informative under real-world conditions.

Turning detection required converting GPS coordinates from latitude and longitude into Cartesian coordinates for accurate geometric and kinematic analysis. The transformations used are shown in equations 1 and 2. Given a point on the Earth’s surface with latitude ∅ and longitude λ, the x and y coordinates were calculated as:

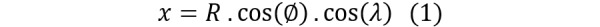

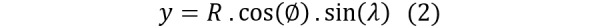





where R is the Earth’s radius (approximately 6371 km), and ∅ and λ are expressed in radians.

**Figure 3:**
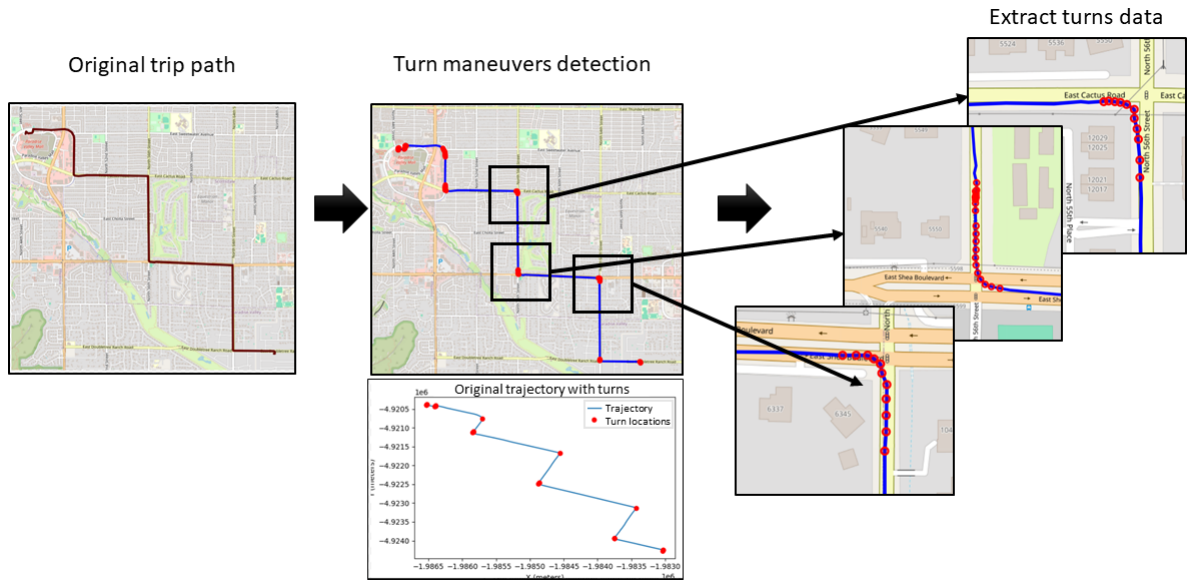
Illustration of the turn maneuver detection process. The leftmost map represents the original trip path recorded using GPS data. The middle section highlights detected turning maneuvers, marked by red dots, which were identified based on changes in trajectory. The rightmost section zooms in on extracted turn segments, showing detailed turning paths.

To identify turning maneuvers, the peak point of each arc path was detected by analyzing the rate of change in x and y rectangular coordinate arrays. By computing the first derivative (dy/dx), the directional changes were captured, with abrupt gradient shifts indicating deviations from straight paths. To filter out gradual curves, various thresholds were tested. It was found that a gradient of 10 degrees (0.174533 radians) reliably captured meaningful turns. However, relying on the gradient alone produced occasional errors. Points where dx=0, resulting in undefined gradients, were discarded. Additionally, turn stamp points with 0 speed, which typically corresponded to stationary periods rather than active maneuvers, were also excluded. Further refinement addressed redundant peak detections within a single turn. In some cases, multiple nearby peaks were incorrectly classified as separate turns. To resolve this, a smoothing approach was applied. Sequential turn points were merged and replaced by their median index, reducing overlap and ensuring 1 peak per turn event. Once peaks were identified, all surrounding points were extracted by applying a temporal window. Based on empirical observations, a turn maneuver was identified by including all sensor readings occurring 15 seconds before and after the peak point. Accelerometer and gyroscope readings were downsampled to align with the 1 Hz GPS rate by averaging every 24 readings from the higher frequency sensors. Figure 3 illustrates an example of turns extracted from a sample trip.

#### Features Engineering (Radial Acceleration)

An additional engineered feature was radial acceleration, which captures how sharply and assertively a driver navigates turns. Higher radial acceleration values may indicate sharper bends or faster speeds, while rapid fluctuations within a maneuver suggest frequent microadjustments in steering and speed. These patterns can reflect reduced motor control and cognitive instability. Radial acceleration was computed by taking the temporal gradient of the gyroscope data along the X, Y, and Z axes. This provided a continuous measure of changes in angular velocity over time, enriching the representation of driver motion. Equations 3-5 show the computation for each axis:

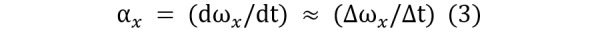

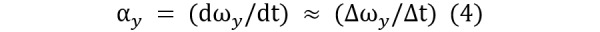

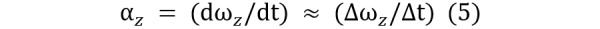







Table 4 summarizes the final set of 10 features used in the input tensor for all models. Each feature represents a specific aspect of motion captured by the GPS, accelerometer, or gyroscope sensors. The Gyro_X_Acc, Gyro_Y_Acc, and Gyro_Z_Acc variables correspond to the angular accelerations derived from the temporal derivatives of the gyroscope signals.

**Table 4:** Final set of sensor-based features used in the dataset. The table lists the 10 input channels used across all experiments. Each feature captures a different dimension of vehicle motion using raw or derived signals from speed, accelerometer, and gyroscope sensors.

Feature or channel	Description
Speed	Vehicle speed measured in meters per second (m/s)
Acc_X	Linear acceleration along the vehicle’s longitudinal axis (forward/backward)
Acc_Y	Linear acceleration along the lateral axis (side-to-side motion)
Acc_Z	Linear acceleration along the vertical axis (up or down motion)
Gyro_X	Angular velocity around the x-axis (roll rate)
Gyro_Y	Angular velocity around the y-axis (pitch rate)
Gyro_Z	Angular velocity around the z-axis (yaw rate or heading change)
Gyro_X_Acc	Angular acceleration around the x-axis, derived from Gyro_X
Gyro_Y_Acc	Angular acceleration around the y-axis, derived from Gyro_Y
Gyro_Z_Acc	Angular acceleration around the z-axis, derived from Gyro_Z

### Multivariate Time Series Data Formulation

This study used 2 datasets: one capturing entire trip readings and another focused on turning maneuvers. Both were formatted as multivariate time series to preserve the sequential nature of driving behavior. Depending on the dataset, each row represented either a full trip or a turn, and each sensor served as a separate time-dependent variable. To standardize input lengths for model training, sequences were aligned and resampled through interpolation, ensuring uniform temporal resolution across samples.

### Time Series Alignment and Interpolation

Trip and turn durations varied widely across drivers, requiring a preprocessing step to standardize time series lengths. Without it, inconsistent sequence sizes would hinder the model’s ability to learn temporal patterns. To address this, interpolation was applied to resample all sequences to a fixed number of time points. To select the optimal length, different resolutions were tested by plotting samples and evaluating the balance between data redundancy and information loss. Alignment and interpolation were applied separately to the trip and turn datasets to maintain consistency within each.

**Figure 4:**
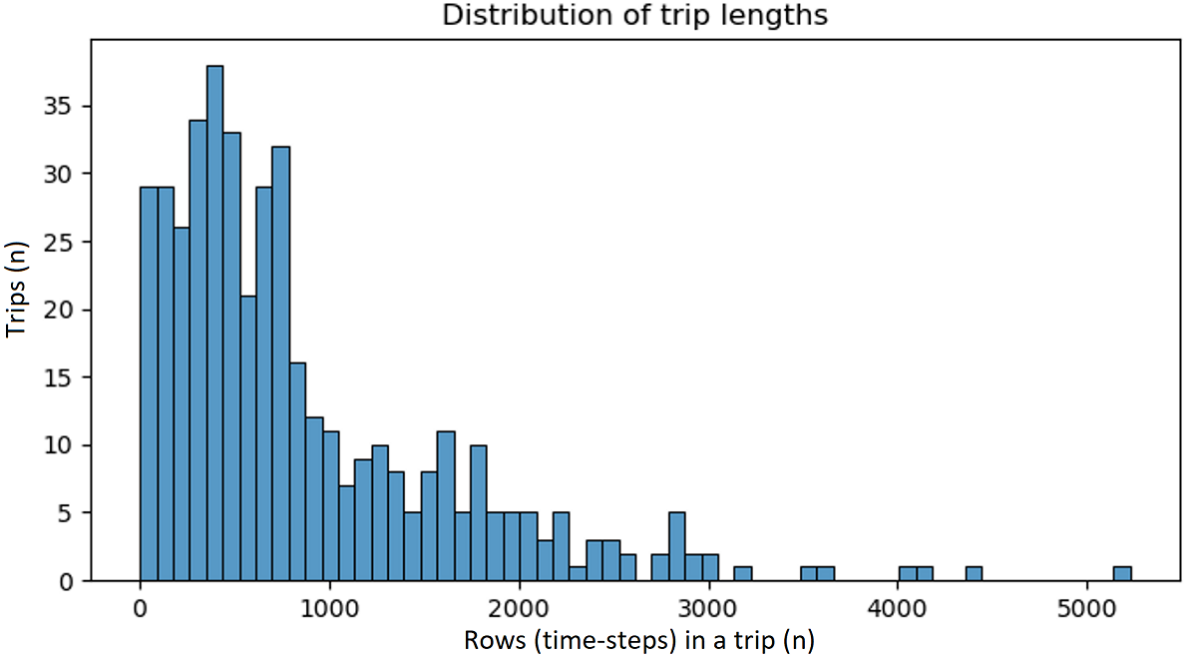
Distribution of trip lengths in the raw dataset. Each bar represents the number of trips with a given sequence length (in time steps). The histogram was used to inform the selection of a standardized interpolation length that balances coverage and temporal resolution.

For the trip dataset, the distribution of raw sequence lengths (Figure 4) was analyzed, and a target that preserved most trips without excessive compression was selected. This choice was further validated by visually inspecting sensor signals before and after resampling, confirming that key temporal patterns were retained. Based on this process, a fixed length of 1200 points was selected for the trip dataset.

For the turning maneuvers dataset, the extraction process described in Turning Maneuvers Data Extraction produced segments with a standardized length of 31-time steps by centering each around the turn midpoint and including a fixed number of points before and after. In most cases, this window was fully available, making interpolation unnecessary. However, in rare edge cases, such as turns at the very start or end of a trip, the available context was limited, resulting in shorter segments. These were interpolated to ensure consistency across the dataset.

### Final Data Representation

**Figure 5:**
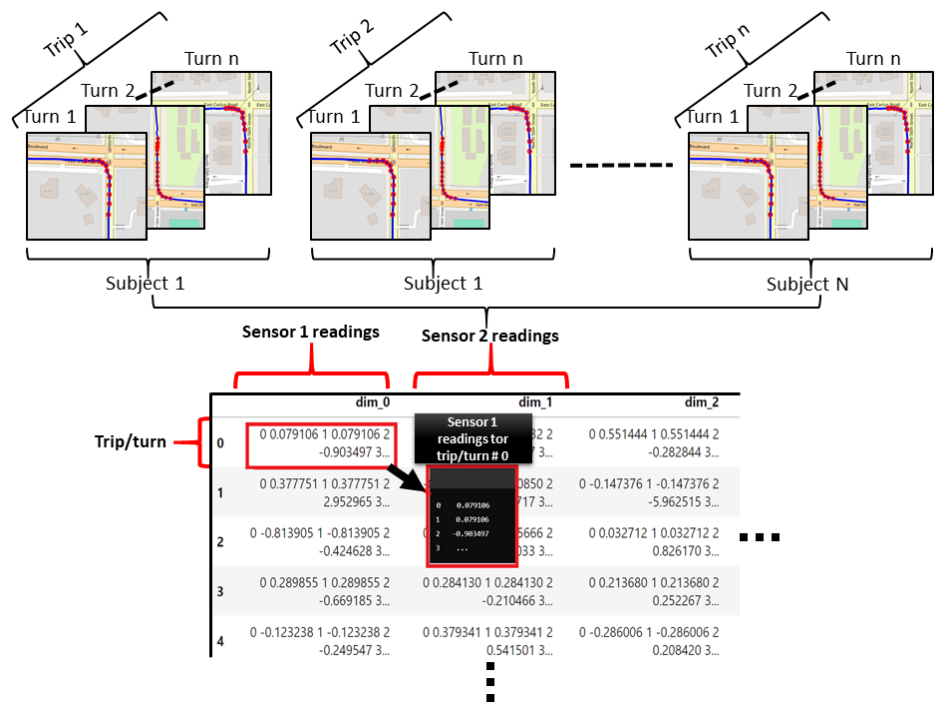
Representation of the MTS dataset structure used. Each row corresponds to either an entire trip or a turn, depending on the dataset. The dataset is formatted as a 3D tensor with dimensions (trip or turn, channels [sensors], and sensor readings). MTS: multivariate time series.

After interpolation, all sensor streams were consolidated into a single structured format. Each trip or turn was represented as a 3D tensor with dimensions N × C × L, where N is the number of trips or turns, C is the number of channels (sensors), and L is the standardized sequence length. Each row corresponds to a distinct trip or turn, columns represent the sensor channels (eg, speed), and the entries hold the sequential readings for the interpolated time steps. Figure 5 shows how all sensor data were aligned and merged into this final format.

### Experiments

This section describes the experimental setup and methods used to analyze driving behavior and classify participants based on cognitive status. We hypothesize that naturalistic driving contains digital signatures that can support early MCI detection. The central question is how to best process and model this data to maximize predictive performance. To explore this, we designed 3 experiments based on the same source of driving data but varying in input representation and modeling strategy. By decomposing the problem along 2 dimensions, what part of the data to use (entire trip vs turns) and how to architect the model (single vs multiple inputs or models), we aim to identify which configurations most effectively preserve behaviorally relevant information.

Three main architecture families were used: 1D convolutional neural networks (1D-CNNs), recurrent neural networks (RNNs), and temporal convolutional networks (TCNs); because they align well with the characteristics of naturalistic driving data, which are multivariate, sequential, and temporally dependent. 1D-CNNs were selected for their ability to detect short, localized motion patterns such as rapid accelerations or turning dynamics; RNNs were included to capture longer behavioral trends that unfold over an entire trip; and TCNs were chosen for their ability to model both short- and long-range dependencies efficiently and robustly. Variants within each family (eg, lightweight 1D-CNNs and attention-based RNNs) were explored to identify which architectural style best captures early MCI-related temporal signatures in real-world driving behavior.

All models were trained using a unified configuration to ensure comparability across experiments. The loss function was standard cross-entropy with class weighting applied when needed to address class imbalance. Standard scaling normalization was applied where indicated in the tables. Optimization was performed with AdamW using a learning rate of 1×10^–3^ and a weight decay of 1×10^–4^. A cosine annealing learning-rate scheduler with T_max_ equal to the number of epochs was used to promote stable convergence. Training was conducted with a batch size of 32 for 200 epochs (reduced from 500 after observing convergence before epoch 200). Dropout was applied only when specified by the architecture variant, as detailed in the appendices.

**Figure 6:**
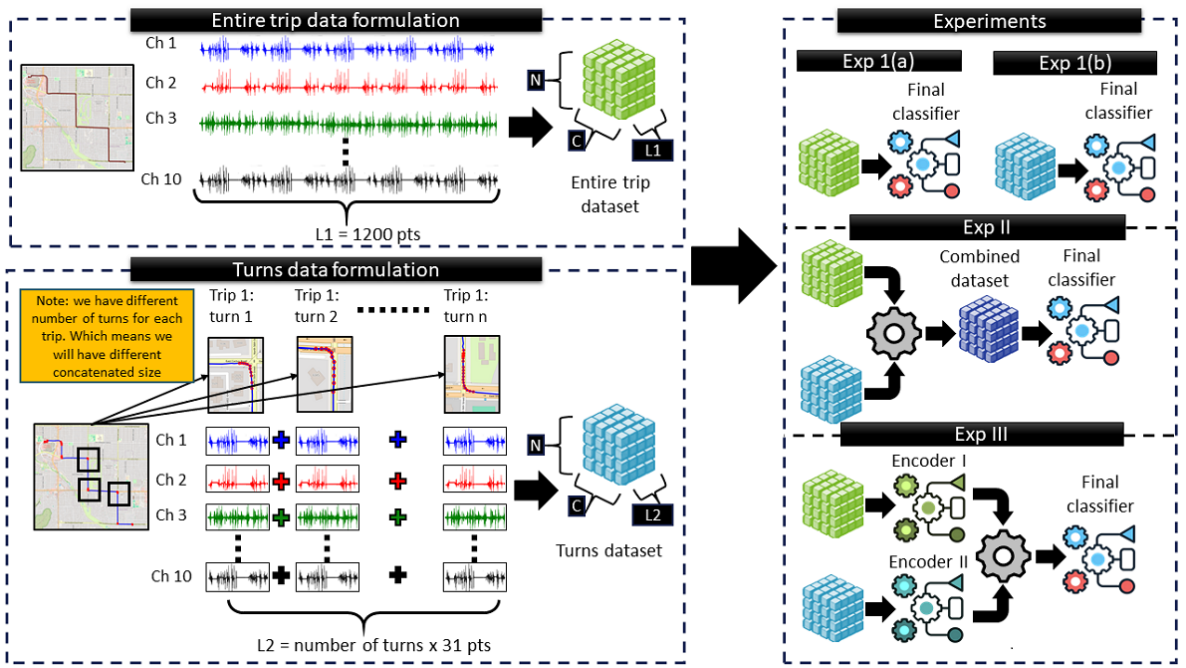
Overview of the data formulation and experimental paradigms. The left side shows the data formulation that produces the 2 multivariate inputs used in this study: the entire-trip sensor readings (green) and concatenated turn-segment readings (blue). The right side summarizes how the produced data are used in our experiment, which consists of 3 modeling paradigms: Exp 1 (single-view models trained on trips or turns separately), Exp 2 (feature-level fusion of the 2 inputs), and Exp 3 (dual encoders with late fusion).

Table 5 summarizes the details of the experiments, while Figure 6 illustrates the modeling strategies in each.

**Table 5:** Summary of experimental setups showcasing the data source, feature representation, and modeling paradigm. Sensor channels include speed, linear accelerations (X/Y/Z), angular velocities (X/Y/Z), and derived angular accelerations (X/Y/Z).

Experiment	Data source	Feature representation (input to model)	Predictive-modeling strategy
1(a)	Entire trip only	Multivariate time series (10 raw sensor channels)	Single-view deep models (1D-CNN^a^, RNN^b^, and TCN^c^ variants)
1(b)	Turning maneuvers only	Multivariate time series (same 10 channels, windowed around turns)	Single-view deep models (1D-CNN, RNN, and TCN variants)
2	Trip and turn signals (concatenated)	Feature-level fusion (20-channel time series)	Single model (1D-CNN, RNN, and TCN) trained on fused input
3	Trip and turn signals (separate)	Two parallel 10-channel time series	Dual encoders with late fusion

^a^1D-CNN: 1D convolutional neural network.

^b^RNN: recurrent neural network.

^c^TCN: temporal convolutional network.

### Experiment 1: Single Data Representation With Single Model

This experiment evaluates the effectiveness of using a single type of input, either full trips or turn segments, modeled independently (Figure 6). All input data was treated as a multivariate time series and interpolated to a fixed length using 10 sensor channels. For each data type, various model architectures were tested, including 1D-CNNs, RNNs, and TCNs (details in Multimedia Appendix 1). In the full-trip setting (experiment 1(a)), each trip was used as a standalone sequence to capture broad driving behavior. In contrast, the turn-only setting (experiment 1(b)) focused on concatenated turn segments. This experiment isolated a specific behavior substructure to examine whether it holds predictive power on its own.

### Experiment 2: Combined Input With Single Model (Data Fusion)

In this experiment, feature-level fusion was explored by combining full-trip and turn-based inputs into a single tensor, as illustrated in Figure 6. Sensor readings from 10 channels were extracted for the entire trip and interpolated to a fixed length of 1200 time points (L₁) to ensure consistency. Separately, all turn segments from each trip were concatenated and interpolated to the same length (L₁=1200), despite variability in the number of turns across trips. After alignment, both sequences were concatenated along the channel dimension, resulting in a unified tensor with shape (N, C=20, and L=1200), where N denotes the number of trips and C includes 10 channels from the trip data and 10 from the turn data. As in experiment 1, a range of model architectures was evaluated, including 1D-CNNs, RNNs, and TCNs, using the fused input. The objective was to assess whether the joint modeling of global and localized driving behavior could improve classification performance. Further architectural details are provided in Multimedia Appendix 2.

### Experiment 3: Separate Inputs and Models With Late Fusion (Model Fusion)

In the final experiment, a model fusion approach was adopted. Separate models were trained independently on the trip-level and turn-level data. As shown in Figure 6, a total of 2 distinct datasets were prepared for each trip. In the upper branch, the complete 10-channel sensor stream was resampled to a fixed length of L₁ points and passed to encoder 1, where a latent representation of global driving behavior was learned. In the lower branch, all turning maneuvers for the same trip were concatenated, and the resulting sequence was standardized to L₂ points (which differed from L₁) before being input to encoder 2.

Encoders were selected from 3 architectural families, 1D-CNN, RNN, or TCN (for example, DualTinyFCN, DualGRU, and DualTCN), and were trained with separate weights. The latent vectors from both encoders were combined using a late fusion classifier. This allowed the model to integrate complementary global and local driving features while maintaining the independence of each encoder. Further model details are provided in Multimedia Appendix 3.

### Model Training Framework

The training framework for all experiments followed a structured procedure. The process began with data preprocessing, where the turning maneuvers dataset was transformed into a 3D tensor representation with dimensions (N, C, and L). To evaluate the model’s performance in distinguishing between cognitively healthy individuals and those with MCI, a 7-fold interparticipant cross-validation strategy was implemented. Given the limited sample size, no independent test set was used. Instead, interparticipant cross-validation was adopted to maximize data use while ensuring that the evaluation remained unbiased. Each fold contained a unique set of participants for testing, with the remaining participants used for training, ensuring that no individual appeared in both sets and thereby eliminating any risk of data leakage across folds.

Stratification was handled at the participant level. Each fold maintained an equal ratio of cognitively normal to MCI participants in the validation set. As the models operated on trip or turn-level data (rather than directly on participants), it was not possible to simultaneously stratify the trip or turn samples and preserve strict participant separation. We therefore prioritized participant-level stratification, as it provides a more reliable measure of generalization to unseen drivers, even though the resulting number of trips or turns per class could become imbalanced across folds. To mitigate any resulting class imbalance, balanced accuracy was reported alongside overall accuracy, area under the receiver operating characteristic curve (AUC), precision, and recall to provide a fairer assessment of performance across both diagnostic groups.

Once the dataset was partitioned, each model was trained on the training folds and evaluated on unseen participants in the validation fold. The average metrics across all 7 folds were used to report the final performance.

### Evaluation Metrics

To assess the performance of the ML models, multiple evaluation metrics were used. Given the class imbalance in the dataset, relying solely on accuracy could be misleading. Therefore, we used a combination of accuracy, balanced accuracy, AUC, precision, and recall to provide a comprehensive evaluation of model effectiveness.

Accuracy measures the overall proportion of correctly classified instances, but can be biased when the dataset is imbalanced. Balanced accuracy addresses this issue by computing the average recall across both classes, making it a more reliable metric for evaluating performance in cases where 1 class is underrepresented. AUC provides an indication of the model’s ability to distinguish between the 2 classes, with higher values indicating better separability. Precision quantifies the proportion of positive classifications that are actually correct, which is particularly important in minimizing false positives. Recall, on the other hand, measures the model’s ability to correctly identify positive instances, ensuring that individuals with MCI are accurately detected. The formulas used for computing these metrics are summarized in Table 6.

**Table 6:** Evaluation metrics and their mathematical formulations.

Metric	Formula^a^	Description
Accuracy	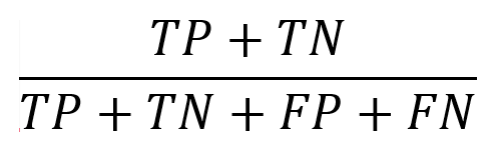	Proportion of correctly classified instances out of all cases.
Balanced accuracy	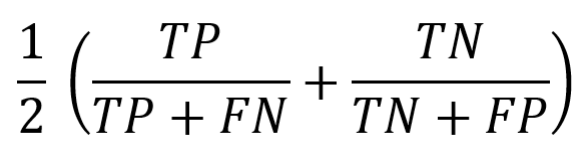	Average recall for both classes, accounting for imbalances.
AUC^b^	Computed from the ROC^c^ curve	Measures the model’s ability to distinguish between classes.
Precision	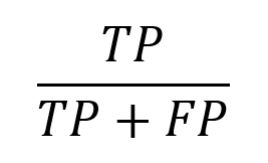	Proportion of correctly identified positive cases among predicted positives.
Recall (sensitivity)	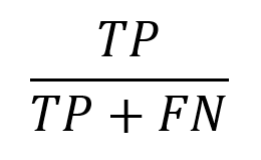	Proportion of actual positives correctly classified by the model.

^a^FN: false negative, FP: false positive, TN: true negative, TP: true positive.

^b^AUC: area under the receiver operating characteristic curve.

^c^ROC: receiver operating characteristic.

### Ethical Considerations

A total of 22 participants were initially recruited, informed about the study, and provided written consent. This study was approved by the Institutional Review Board at Arizona State University (STUDY00006547).

Participant privacy and confidentiality were strictly maintained throughout this study. All data were deidentified using unique participant identification codes, and no personally identifiable information was included in any analyses or publications.

Participants received no financial compensation for their participation.

## Results

This section reports the performance of the 3 experimental paradigms, single-view modeling (experiment 1), feature-level data fusion (experiment 2), and model-level fusion (experiment 3), evaluated with 7-fold, participant-stratified cross-validation. For every experiment, we averaged accuracy, balanced accuracy, AUC, precision, recall, and *F*_1_-score across folds.

### Experiment 1 Results

Experiment 1 examined whether early MCI signals could be more effectively captured from full-trip data or from isolated turning maneuvers when modeled independently. To provide a performance reference, a nontemporal baseline model was first implemented using summarized trip-level statistics from the raw sensor data. For each trip, the mean and SD of the accelerometer (X, Y, and Z), gyroscope (X, Y, and Z), and speed signals were computed, resulting in 14 static features. These features were used to train a random forest classifier, representing a simple benchmark based on conventional telematics features used in earlier driving and cognitive-monitoring studies [19,25]. Table 7 summarizes the cross-validated results for 21 single-view models. The full-trip experiment (1(a)) consistently outperformed the turn-only experiment across all tested architectures. The best performance was achieved by a lightweight convolutional model (TinyFCN without normalization but with class weighting), reaching 78.3% accuracy, 73.6% balanced accuracy, and an AUC of 76.7%. Other strong performers included deeper convolutional models (InceptionTimeSmall) and attention-enhanced recurrent networks (gated recurrent unit, GRU + Attn), suggesting that both paradigms can leverage the extended temporal context of full trips. Channel normalization was found to make results worse.

**Table 7:** Cross-validation results for all experiments. Metrics are averaged over 8 folds.

Experiment	Model	Normalize	Weights	Accuracy (%)	BA^a^ (%)	AUC^b^ (%)	Precision (%)	Recall (%)	*F*_1_-score (%)
Baseline	RF^c^	Yes	—^d^	55	49	52	33	20	25
1(a)	1D-CNN^e^	No	No	73	63	62	57	72	63
1(a)	GRU^f^	No	Yes	73	69^g^	73^g^	63	75	60
1(a)	GRU and attention	No	Yes	77	69	66	64	79	69^g^
1(a)	GRU and multiattention	No	Yes	71	69	65	57	83^h^	64
1(a)	Inception lite	Yes	Yes	59	61	56	47	80	54
1(a)	Inception lite	No	Yes	68	65	60	63	64	53
1(a)	InceptionTimeSmall	Yes	Yes	69	69	69	65^h^	67	58
1(a)	InceptionTimeSmall	No	Yes	78^g^	66	64	69^g^	71	65^h^
1(a)	LSTM^i^	No	Yes	69	69	64	56	78	59
1(a)	ResNet TSC^j^	Yes	Yes	65	66	64	52	82	60
1(a)	ResNet TSC	No	Yes	77^h^	67	65	57	73	63
1(a)	TCN^k^	No	Yes	71	60	59	52	68	57
1(a)	TCN and attention	No	Yes	58	58	62	47	86^g^	56
1(a)	TinyFCN	Yes	No	73	63	62	57	72	63
1(a)	TinyFCN	Yes	Yes	72	66	64	65	73	62
1(a)	TinyFCN	No	Yes	78^g^	74^g^	77^g^	65^h^	66	64
1(a)	Tiny TCN	Yes	Yes	63	64	65	51	76	57
1(a)	Tiny TCN	No	Yes	64	60	58	48	82	57
1(b)	1D-CNN	No	No	63	54	53	44	30	34
1(b)	TinyFCN	Yes	Yes	63	56	58	44	53	46
1(b)	TinyFCN	No	Yes	59	57	59	42	64	47

^a^BA: balanced accuracy.

^b^AUC: area under the receiver operating characteristic curve.

^c^RF: random forest.

^d^Not available.

^e^1D-CNN: 1D convolutional neural network.

^f^GRU: gated recurrent unit.

^g^The second best values.

^h^The best values.

^i^LSTM: long short-term memory.

^j^TSC: time series classification.

^k^TCN: temporal convolutional network.

In contrast, the turn-only experiment 1(b) showed significantly lower performance. The best model in this setting, TinyFCN with normalization, reached only 62.7% accuracy and 56.3% balanced accuracy, a decline of about 16 percentage points compared to its trip-level counterpart. Precision dropped below 45% in most turn-only models, indicating insufficient discriminative power in short maneuver windows. Some architectures (eg, TCN + Attn) achieved high recall (up to 86.4%) by overpredicting the positive class, but at the cost of precision and balanced accuracy. Due to the weak performance of turn-only models, subsequent experiments focused on configurations that included turn data only in combination with full-trip data, either through feature-level fusion (experiment 2) or model-level fusion (experiment 3). This decision avoided further computational investment in a setting that showed limited utility for early AD detection under naturalistic conditions.

Overall, experiment 1 demonstrates that global driving behavior across full trips offers substantially more predictive information than isolated turning maneuvers. Among all architectures, compact convolutional models such as TinyFCN provided the best balance of performance and efficiency, while recurrent models with attention were also competitive. The performance of full-trip TinyFCN (accuracy ≈78%, AUC ≈77%) serves as the baseline for subsequent fusion experiments.

It is important to note that earlier studies [5,6] identified turn-specific kinematic features as promising biomarkers for MCI. However, those findings were obtained in controlled environments, where participants drove short, predefined routes under standardized conditions. In such settings, turn segments are clearly defined, confounding variables are minimized, and behavioral comparisons are more reliable. In contrast, the present study analyzed turning maneuvers within naturalistic, long-distance trips that varied widely in road conditions, traffic, and route design. As a result, turn segments were shorter, noisier, and occasionally truncated by sensor limitations. These factors contributed to the limited performance of turn-only models and motivated the shift toward combining turn data with richer trip-level context in the remaining experiments.

### Experiment 2 Results

In experiment 2, trip and turn signals were concatenated at the feature level, and a single model was trained on the resulting 20-channel input. A total of 4 architectures (TinyFCN, GRU, long short-term memory, and TCN) were evaluated. Their cross-validated performance is reported in Table 8.

**Table 8:** Cross-validated performance of feature-level data-fusion models (experiment 2). Trip and turn signals were concatenated into a single 20-channel input and evaluated with 7-fold participant-stratified cross-validation.

Experiment	Model	Normalize	Weights	Accuracy (%)	BA^a^ (%)	AUC^b^ (%)	Precision (%)	Recall (%)	*F*_1_-score (%)
2	TinyFCN	No	Yes	62	59	52	44	86^c^	56^c^
2	GRU^d^	No	Yes	68^e^	63	63^e^	52^e^	62	53
2	LSTM^f^	No	Yes	67	67^c^	64^c^	55^c^	69^e^	55^e^
2	TCN^g^	No	Yes	70^c^	63^e^	63	50	62	53

^a^BA: balanced accuracy

^b^AUC: area under the receiver operating characteristic curve.

^c^The best values.

^d^GRU: gated recurrent unit.

^e^The second best is only underlined.

^f^LSTM: long short-term memory.

^g^TCN: temporal convolutional network.

Overall, performance improved over the turn-only models from experiment 1(b), confirming that global trip context can offset the sparsity of turn data. However, none of the fusion models outperformed the best full-trip baselines from experiment 1(a). The highest accuracy (70%) was achieved by TCN, though its AUC (63%) and balanced accuracy (63%) fell short of the full-trip TinyFCN benchmark. Long short-term memory delivered the highest balanced accuracy (67%), while GRU achieved similar accuracy and recall but lower precision. TinyFCN, which had previously performed best on full trips, dropped to 62% accuracy and 52% AUC, suggesting that feature-level concatenation may impair its ability to model trip dynamics effectively.

These results indicate that early fusion does not offer a reliable method for combining localized and global driving features. While a modest gain in recall was observed, it came at the cost of precision and overall discriminative power. This degradation likely results from the mismatch in temporal characteristics between trips and turns when merged into a single input. These findings support the need for a late-fusion strategy, as explored in experiment 3, where each data type is modeled independently before integration.

### Experiment 3 Results

In the final experiment, the trip and turn streams were treated as complementary modalities. Each was processed by a dedicated encoder, and their latent vectors were merged in a late-fusion classifier. Table 9 summarizes the cross-validated results for 7 dual-branch architectures. The dual-encoder strategy outperformed the feature-level fusion models from experiment 2 across all metrics and approached the performance of the best full-trip baselines from experiment 1(a). The highest overall accuracy (75%) was achieved by DualTinyFCN, which also recorded the best AUC (68%) and matched the highest recall (74%). DualTCN with self-attention produced the strongest balanced accuracy (72%) and recall (77%), while maintaining 75% accuracy. DualGRU with a single attention head offered a solid alternative at 73% accuracy and 65% balanced accuracy; its multihead variant improved recall to 84% but at the cost of precision.

**Table 9:** Cross-validation results for model-level fusion (experiment 3). Each architecture comprises separate trip and turn encoders whose outputs are combined by either concatenation (DualTinyFCN) or a learned gating mechanism (all others).

Experiment	Model	Normalize	Weights	Accuracy (%)	BA^a^ (%)	AUC^b^ (%)	Precision (%)	Recall (%)	*F*_1_-score (%)
3	Dual GRU^c^	No	Yes	63	62	58	47	67	50
3	Dual GRU Attn	No	Yes	73	65^d^	63	56^d^	76	62^d^
3	Dual GRU MultiHead	No	Yes	66	63	66	52	84^e^	60
3	Dual LSTM^f^	No	Yes	65	62	64	51	61	50
3	DualTCN	No	Yes	66	60	67^d^	47	79^d^	57
3	DualTCN Attn	No	Yes	75^d^	72^e^	66	57^e^	77	63^e^
3	Dual Tiny FCN^g^	No	Yes	75^e^	65	68^e^	53	74	61

^a^BA: balanced accuracy

^b^AUC: area under the receiver operating characteristic curve.

^c^GRU: gated recurrent unit.

^d^The second best values.

^e^The best values.

^f^LSTM: long short-term memory

^g^FCN: fully convolutional network

Relative to experiment 2, balanced accuracy improved by 6 to 8 percentage points, and AUC increased by about 5 points, suggesting that separate feature extraction for trips and turns reduced the interference observed with early fusion. While none of the dual-branch models surpassed the single-view TinyFCN baseline from experiment 1(a), the performance gap was reduced to within 4 points, with noticeably stronger recall and class balance.

These results indicate that turning behavior carries useful complementary information, which becomes effective when modeled independently before fusion. However, the late-fusion models still fell short of the trip-only baseline, unlike in earlier studies conducted under controlled driving conditions. A likely explanation is the greater behavioral variability in naturalistic turn maneuvers, where factors such as traffic, shallow bends, truncated segments, and GPS inconsistencies introduce noise that diminishes signal quality. To overcome this, 2 improvements are proposed. First, a more selective turn-extraction method could be implemented to filter out low-information maneuvers (eg, shallow curves or stop-sign rolls) and retain only cognitively demanding turns. Second, the use of an adaptive fusion mechanism, such as a trainable gating or context-aware attention layer, could allow the model to dynamically weigh turn features based on context, rather than relying on fixed integration. These enhancements may help future late-fusion models surpass the trip-only benchmark.

### Participant-Level Performance of the Best-Performing Model

**Figure 7:**
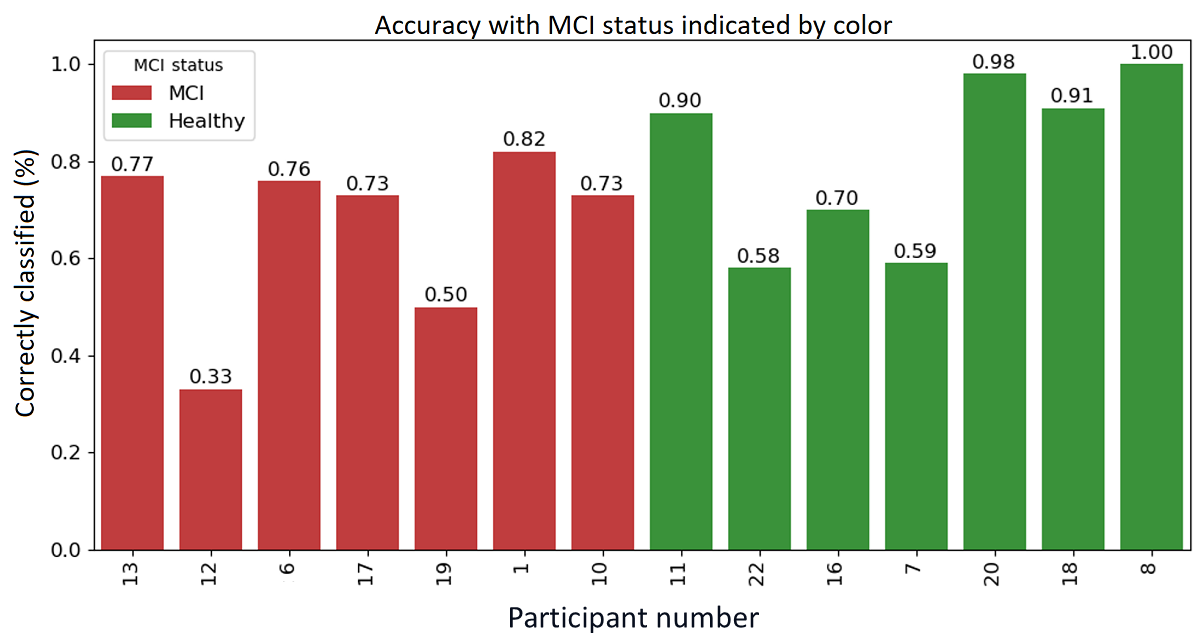
Per-subject trip classification accuracy of the best performance model, color-coded by cognitive status (green=healthy, red=MCI). MCI: mild cognitive impairment.

To further evaluate model effectiveness, participant-level performance was analyzed using the best-performing model from experiment 1(a), which achieved the highest average accuracy (78%). Figure 7 shows the percentage of correctly classified trips per participant, color-coded by diagnostic group.

This analysis revealed that trips from cognitively healthy participants were classified more accurately than those from the MCI group. Within the MCI cohort, classification accuracy appeared to correlate with the number of available trips. Participants 12 and 19, who contributed only 3 and 4 trips respectively, showed the lowest accuracies (33% and 50%). In contrast, participant 1, also in the MCI group, provided 28 trips and achieved over 80% accuracy, suggesting that greater data volume improves participant-specific classification performance. These findings support the decision to evaluate trips individually rather than applying a majority-vote rule per participant, as done in previous work. As MCI symptoms tend to appear episodically, a majority-vote approach may misclassify participants with sporadic impairments. For instance, participant 12, clinically diagnosed with MCI, was misclassified on 2 of 3 trips. A majority-vote method would incorrectly label this participant as healthy, masking the sporadic but clinically meaningful deviations this framework aims to detect.

The granular trip-wise approach captures the variability that defines mild impairment and provides a more sensitive early warning mechanism than binary participant-level labels. This variability can be expressed as a frequency-based risk score based on the proportion of trips classified as impaired for each driver. Rather than producing a fixed “MCI” or “healthy” label, the score outputs a continuous risk value between 0 and 1, allowing clinicians to define thresholds for follow-up assessment. The choice of such a threshold will have practical implications. A low threshold (eg, flagging drivers if 10% or more of their trips are classified as impaired) increases sensitivity and supports early detection but may lead to more false positives and unnecessary follow-up. A high threshold (eg, 50%) reduces false positives but risks missing individuals with sporadic lapses (precisely the pattern seen in early MCI). As the risk score is continuous, thresholds can be tailored to specific clinical contexts. For example, high sensitivity may be prioritized in population-level screening, while higher precision may be preferred in specialist clinics. By presenting performance curves across thresholds or reporting AUC values alongside confusion matrices at clinically relevant cutoffs, these trade-offs can be made explicit and support informed deployment of the model in practice.

Trip count was also found to influence the stability of those episodic signals. With fewer than 5 trips, a single misclassified journey can flip a driver’s apparent status, explaining the wide swings in accuracy for participants 12 and 19. As the dataset grows (through additional participants, extended monitoring, or data augmentation), the law of large numbers should attenuate this volatility and sharpen the distinction between truly unimpaired drivers and those with early cognitive change. Overall, these results reinforce the importance of context-aware, trip-level evaluation for detecting early MCI. Rather than relying on binary labels, future implementations should adopt frequency-based risk scoring to translate trip-level outputs into longitudinal, clinically actionable indicators.

## Discussion

This study demonstrated the potential of naturalistic driving data as a digital biomarker for early MCI detection. Prior limitations, such as controlled test settings, reliance on summary statistics, and rigid evaluation protocols, were addressed through a framework that embraces real-world variability and context-aware analysis, structured around the 3 research questions posed in the Introduction section.

First, the robustness of driving-based biomarkers under naturalistic conditions was assessed (RQ1). Models trained on full-trip data achieved 78% accuracy (AUC 77%) despite variations in routes, traffic, and sensor noise, while turn-only inputs underperformed by approximately 16 percentage points. These results affirm that early cognitive change is detectable through naturalistic driving; however, short turning maneuvers alone were found to be too noisy to provide reliable indicators.

Second, to address RQ2, a multistage pipeline was developed to transform raw, noisy, multisensor data into structured model inputs. Signals of varying frequencies were aligned, trips segmented, and interpolation applied to ensure consistency. Turn maneuvers were extracted using a targeted method, yielding maneuver-level data while preserving temporal and sensor fidelity for deep learning.

Third, modeling strategies were compared under current data limitations (RQ3). Single-view models on full-trip data remained strongest. While early fusion of trip and turn data failed to outperform this baseline, late fusion using dual encoders significantly improved balanced accuracy and recall, suggesting that turn cues become useful when learned in a dedicated branch. As larger, cleaner turn datasets are acquired, late fusion is expected to surpass trip-only models, especially with mechanisms that emphasize turns only when informative.

In addition, the participant-level analysis revealed that most misclassifications occurred among MCI drivers with few recorded trips, underscoring the effects of behavioral noise and data scarcity. As MCI is marked by sporadic impairments, traditional aggregate predictions may miss subtle signs. By evaluating each trip and maneuver individually, the proposed approach enables detection of episodic lapses, offering a practical early warning system for clinical follow-up.

Future improvements should focus on refining turn extraction through context-aware filtering, enhancing late-fusion models with adaptive gating or attention mechanisms, and enriching the dataset via increased participation and targeted augmentation. Prospective validation and integration of complementary modalities (eg, eye-tracking and in-cabin audio) may yield a more comprehensive behavioral signature of early cognitive decline. Furthermore, given the relatively small dataset, future work could explore the use of transfer learning from models pretrained on large-scale sensor-based time-series datasets. Models trained on motion, activity recognition, or physiological sensing data capture general temporal and kinematic patterns that overlap with vehicle dynamics such as acceleration and rotation. Fine-tuning such pretrained encoders on naturalistic driving data could improve feature extraction, reduce dependence on large labeled cohorts, and enhance generalization to new drivers or sensing platforms. Incorporating transfer learning in this way would be a practical step toward building more data-efficient and scalable cognitive monitoring systems. Collectively, these efforts may further establish naturalistic driving as a viable and nuanced platform for proactive cognitive health monitoring.

## Data Availability

The datasets presented in this paper are not readily available because the data are part of an ongoing study. Requests to access the datasets should be directed to the corresponding author. The underlying code for this study (and training or validation datasets) is not publicly available but may be made available to qualified researchers on reasonable request to the corresponding author.

## Appendix

Multimedia Appendix 1 Experiment 1.

Multimedia Appendix 2 Experiment 2.

Multimedia Appendix 3 Experiment 3.

## Abbreviations

1D-CNN: 1D convolutional neural network
AD: Alzheimer disease
AUC: area under the receiver operating characteristic curve
AWS S3: Amazon Web Services Simple Storage Service
GRU: gated recurrent unit
MCI: mild cognitive impairment
ML: machine learning
OBD: on-board diagnostics
RNN: recurrent neural network
TCN: temporal convolutional network
