# Supplementary material for: Leveraging Naturalistic Driving Digital Biomarkers for Early Mild Cognitive Impairment Detection: Deep Learning Strategies
Source: JMIR Med Inform. 2026 Mar 6;14:e83622. doi: 10.2196/83622 (PMC13005058; doi:10.2196/83622)
Supplement: Multimedia Appendix 1 [file medinform_v14i1e83622_app1.docx]

Exp I Model Architectural Details

This appendix provides a detailed summary of all model variants used in the experiments, including model category, architecture description, parameter count, illustrative figures, and references to prior literature or custom contributions.

| **Model Variant** | **Model Type** | **Description** |
| --- | --- | --- |
| 1DCNN | CNN | 1D convolutional model with stacked layers for temporal feature extraction. |
| TinyFCN | CNN | Three-layer FCN with kernel sizes (8,5,3), ReLU, BatchNorm, and global average pooling. |
| ResNetTSC | CNN | Three ResBlocks with conv pairs (k=5), skip connections, ReLU, and adaptive pooling. |
| Inception Lite | CNN | Two InceptionLite blocks using separable convs with kernels (10,20,40), pooling branch. |
| InceptionTimeSmall | CNN | Two full Inception blocks with bottlenecks, multi-kernel branches, and pooling. |
| GRU | RNN | Single GRU layer (64-128 units), final hidden state to classifier. |
| GRU + Attention | RNN | GRU + single-head attention on sequence output. |
| GRU + MultiAttention | RNN | GRU + multi-head attention (4-8 heads), concatenated output. |
| LSTM | RNN | Single or stacked LSTM layers, using final or pooled output for classification. |
| TCN | TCN | Stack of 4 TCN blocks with dilated causal convs (dilations: 1,2,4,8), residuals. |
| TCN + Attention | TCN | TCN as above, with attention over output sequence. |
| TinyTCN | TCN | Four-layer TinyTCN with dilated causal convs and dropout (p=0.1). |
