# Supplementary material for: Leveraging Naturalistic Driving Digital Biomarkers for Early Mild Cognitive Impairment Detection: Deep Learning Strategies
Source: JMIR Med Inform. 2026 Mar 6;14:e83622. doi: 10.2196/83622 (PMC13005058; doi:10.2196/83622)
Supplement: Multimedia Appendix 2 [file medinform_v14i1e83622_app2.docx]

The table below details the four baseline architectures, TinyFCN, GRU, LSTM, and TCN, used to model each individual data representation in our experiments. Architectural diagrams are provided in the supplementary figures referenced in the last column.

| **Model** | **Family** | **Key Architectural Details** |
| --- | --- | --- |
| TinyFCN | CNN | 3 Conv1D layers (k=8,5,3; channels 128→256→128), BatchNorm, ReLU, Dropout 0.2, AdaptiveAvgPool1d, 128‑d projection |
| GRU | RNN | 2‑layer bidirectional GRU (hidden 128), final hidden concat, Linear classifier |
| LSTM | RNN | 2‑layer bidirectional LSTM (hidden 128), final hidden concat, Linear classifier |
| TCN | Dilated CNN | 4 Temporal Blocks (k=3; dilations 1,2,4,8) with residuals, AdaptiveAvgPool1d, Linear classifier |
