# Supplementary material for: Leveraging Naturalistic Driving Digital Biomarkers for Early Mild Cognitive Impairment Detection: Deep Learning Strategies
Source: JMIR Med Inform. 2026 Mar 6;14:e83622. doi: 10.2196/83622 (PMC13005058; doi:10.2196/83622)
Supplement: Multimedia Appendix 3 [file medinform_v14i1e83622_app3.docx]

This appendix lists the dual‑encoder architectures employed in Experiment IV (model fusion). Each model has two independent branches, one processing full‑trip data and the other processing turn‑only data, followed by a late‑fusion classifier.

| **Dual Model** | **Per-Branch Encoder** | **Fusion Strategy** | **Final Classifier** |
| --- | --- | --- | --- |
| DualGRU | Bidirectional GRU, 2 layers × 128 units | Concatenation → 512 d | 64 → 2 |
| DualGRU + Attn | Bi‑GRU (as above) with single‑head additive attention | Learned scalar gate α·f_trip + (1-α)·f_turn | 64 → 2 |
| DualGRU + MultiAttn | Bi‑GRU with 4‑head self‑attention | Gated fusion (α learned per sample) | 64 → 2 |
| DualLSTM | Bidirectional LSTM, 2 layers × 128 units | Gated fusion | 64 → 2 |
| DualTCN | Temporal‑CNN, 4 dilated blocks (k = 3, d = 1-8) | Gated fusion | 64 → 2 |
| DualTCN + Attn | Same TCN backbone + self‑attention block | Gated fusion | 64 → 2 |
| DualTinyFCN | TinyFCN (Conv k = 8‑5‑3, 64 filters) | Concatenation → 256 d | 64 → 2 |
